# New Sesquiterpenoids and a Diterpenoid from *Alpinia oxyphylla*

**DOI:** 10.3390/molecules20011551

**Published:** 2015-01-16

**Authors:** Lei Hou, Gang Ding, Baolin Guo, Wenhua Huang, Xiaojian Zhang, Zhiyong Sun, Xiangfen Shi

**Affiliations:** 1 Key Laboratory of Bioactive Substances and Resources Utilization of Chinese Herbal Medicine, Ministry of Education, Institute of Medicinal Plant Development, Chinese Academy of Medical Sciences & Peking Union Medical College, Beijing 100193, China; E-Mails: HL1215mj@163.com (L.H.); blguo@implad.ac.cn (B.G.); 2 The First Affiliated Hospital of Zhengzhou University, Zhengzhou 450052, China; E-Mails: zhangxj1130@163.com (X.Z.); szy0678@126.com (Z.S.); xiangfenshi@163.com (X.S.)

**Keywords:** *Alpinia oxyphylla*, sesquiterpenoids, diterpenoid

## Abstract

The new compounds 2-methyl-6-isopropyl-7-hydroxymethyl naphthalene (**1**), oxyphyllenone H (**2**), *epi*-oxyphyllenone (**6**), (*E*)-labda-12,14-dien-15(16)-olide-17-oic acid (**3**), and two new natural products **4** and **5** were isolated from the ethyl acetate part of 95% ethanol extract of *Alpinia oxyphylla*, together with six known compounds **7**–**12**. The inhibitory effects of compounds **1**–**12** on α-glucosidase were evaluated, and compounds **1**, **3** and **6** showed moderate bioactive effect, with inhibitory rates of 10.3%, 10.0% and 11.5%, respectively, compared to the positive control acarbose (41.9%) at 20 µg/mL.

## 1. Introduction

**Figure 1:**
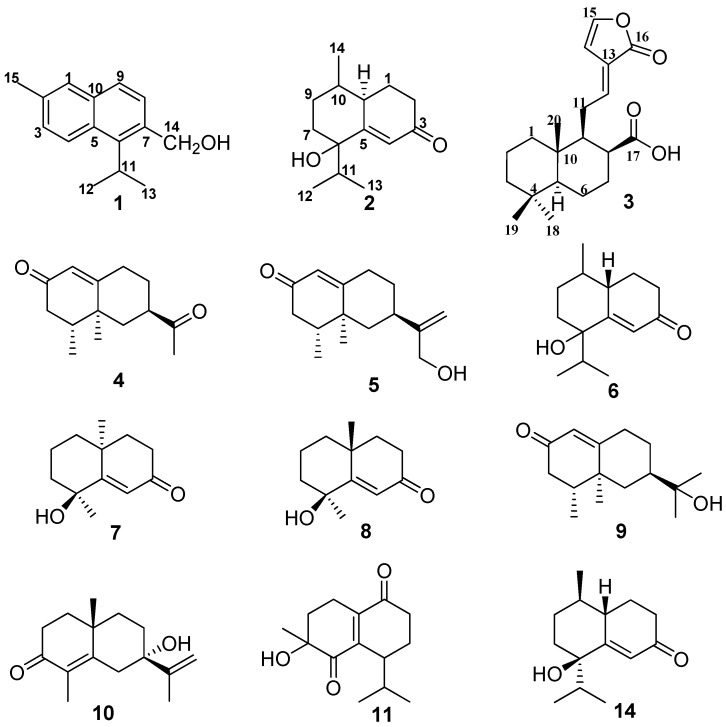
Compounds **1**–**11** from *Alpinia Oxyphylla*.

*Alpinia Oxyphylla* Fructus is the dry ripe fruit of *Alpinia oxyphylla* Miq. (Zingiberaceae), which is widely distributed in South China. It has been used in Traditional Chinese Medicine (TCM) for the treatment of intestinal disorders, dieresis and dementia [1]. Previous phytochemical investigations of this medicinal plant have resulted in the isolation and identification of a series of sesquiterpenoids [2,3,4,5,6]. As part of our ongoing work on the discovery of new active secondary metabolites from this plant, four new compounds, namely 2-methyl-6-isopropyl-7-hydroxymethyl naphthalene (**1**), oxyphyllenone H (**2**), and *epi*-oxyphyllenone (**6**), the stereoisomer of synthesized compound (**14**) [7], (*E*)-labda-12,14-dien-15(16)-olide-17-oic acid (**3**), two new natural products, a diketone **4**, which we have named oxyhylladiketone [8], and (+)-(4*R*,5*S*,7*R*)-13-hydroxynootkatone (**5**) [9], and six known compounds teuhetenone A(**7**) [10], (4S*,5*E*,10R*)-7-oxo-tri-nor-eudesm-5-en-4β-ol (**8**) [11], 11-hydroxyvalenc-1(10)-en-2-one (**9**) [12], (4a*S*,7*S*)-7-hydroxy-1,4a-dimethyl-7-(prop-1-en-2-yl)- 4,4a,5,6,7,8-hexahydronaphthalen2(3*H*)-one (**10**) [5], oxyphyllone E (**11**) [13] and chrysin-7-*O*-(β-d-glycopyranoside) (**12**) [14] (Figure 1) were isolated from the ethyl acetate fraction of a 95% ethanol extract of *Alpinia oxyphylla*. Herein we reported the isolation and the structure elucidation of the new compounds and the inhibitory effects of all these compounds on α-glucosidase.

## 2. Results and Discussion

**Figure 2:**
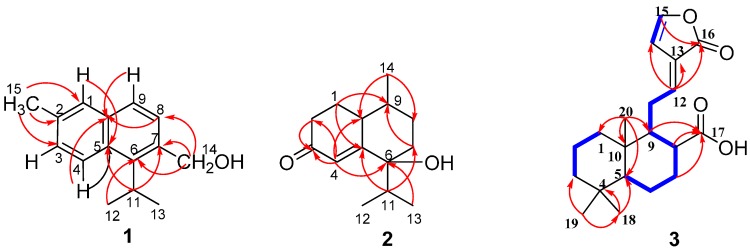
Key COSY and HMBC correlations of compounds **1**–**3**.

Compound **1** was isolated as a pale yellow oil, and the molecular formula was established by HR-EI-MS as C_15_H_18_O (*m*/*z* 214.1347 [M]^+^), indicating seven degrees of unsaturation. The ^1^H-, ^13^C- and HMQC spectra (Table 1) revealed the presence of an isopropyl group [δ_H_ 1.38 (6H, d, *J* = 6.6 Hz), 3.75 (1H, heptet, *J* = 6.6 Hz), δ_C_ 24.15, 29.66], a methyl [δ_H_ 2.54 (3H, s), δ_C_ 22.24] and an oxymethylene [δ_H_ 5.01 (2H, s), δ_C_ 63.81]. Five aromatic protons signals were deduced to a naphthalene ring fragment. The remaining connectivity was solved by HMBC correlations. In the HMBC experiment (Figure 2), The correlations from H-1 to C-3, C-5 and C-10, from H-4 to C-5, C-6 and C-10, from H-8 to C-6, C-7 and C-10, from H-9 to C-5, C-7 and C-10 confirmed the existence a naphthalene ring; the long-range correlations from CH_3_-15 to C-1, C-2 and C-3 confirmed that the methyl group was anchored at C-2; the HMBC correlations from -CH_2_-14 to C-6, C-7 and C-8 determined the direct connection between the C-14 and C-7; the correlations of H-11 with C-5, C-6 and C-7, of CH_3_-12/13 with C-6 displayed that the isopropyl group was connected with C-6. Thus the structure of **1** was determined to be as shown in Figure 1, which was named as 2-methyl-6-isopropyl-7-hydroxymethyl naphthalene.

Compound **2** was also isolated as a colorless oil, and the molecular formula was established by HR-ESI-MS as C_14_H_2__2_O_2_ (*m*/*z* 223.1682 [M+H]^+^), indicating the presence of four degrees of unsaturation. Analysis of the ^1^H-, ^13^C- and HMQC spectra (Table 1 and Table 2) revealed that there were three methyl units, four methylene units, three methines, an oxygenated quaternary carbon, and one α,β-unsaturated carbonyl group [δ_C_ 199.97 (C-3), 122.51 (C-4) and 170.10 (C-5)]. These signals explained two degrees of unsaturation, and implied that there were two rings in the structure of **2**. In the HMBC experiment (Figure 2), the long-range correlations from H-1 to C-2, C-3, C-5, C-9 and C-10, from H-4 to C-2, C-3, C-5, C-6, and C-10 revealed the presence of a cyclohex-2-enone ring system in **2**; the correlations from CH_3_-14 to C-8,C-9 and C-10, from H-11 to C-5, C-6, C-7, and from H-7 to C-9, H-8 to C-6, implied that compound **2** contained one 1-isopropyl-4-methylcyclohexane ring fragment, which was fused with the cyclohex-2-enone ring at C-5 and C-10. Finally, the planar structure of **2** was determined. Analysis of the NMR data, especially the 2D-NMR, revealed that compound **6** also possessed the same planar structure as **2**.

Though there existed distinct differences in the NMR spectra between **2** and **6**, it was very difficult to analyze the relative configuration of compound **2**/**6** by NOESY correlation or by analyzing the coupling constant because of their significantly overlapped signals in the complex ^1^H-NMR spectra. The absolute configurations of C-10 in compound **2**/**6** were determined by CD spectroscopy. The CD spectra of **2** displayed a positive Cotton effect at 320 nm and a negative one at 251 nm, which were completely identical with that of (−)-(1*R*,7*S*,10*R*)-1-hydroxy-11-norcadinan-5-en-4-one, whereas the CD spectra of **6** was nearly opposite to that **2**, thus the stereochemistry of C-10 in **2**/**6** were determined to be the *R*/*S* configuration [15]. In addition, the stereoisomer **14** of **2**/**6** was always synthesized mixed with its isomer, and its structure was postulated from its ^1^H-NMR and mass spectra [7]. This is the first report of the isolation from a natural source of pure stereoisomers of **14**, which were unambiguously characterized by NMR and mass spectroscopy experiments.

**Table 1:** The ^1^H-NMR (600 MHz) spectroscopic data for compounds **1**–**3** and **6**.

No.	1(MeOD)	2(CDCl_3_)	3(CDCl_3_)	6(CDCl_3_)
	δ_H_ (*J* in Hz)	δ_H_ (*J* in Hz)	δ_H_ (*J* in Hz)	δ_H_ (*J* in Hz)
1	7.95, s	1ɑ, 1.74, m	1ɑ, 1.50, m	1ɑ, 1.79, m
		1β, 2.23, m	1β, 1.79, m	1β, 2.17, m
2		2ɑ, 2.26, m	2ɑ, 1.37, m	2ɑ, 2.28, m
		2β, 2.38, m	2β, 1.55, m	2β, 2.42, m
3	7.37, d (7.8)		1.10, 1H,m,3ɑ	
			1.28, 1H,m,3β	
4	7.35, d (7.8)	6.23, s		5.94, s
5			0.97, dd (13.2, 2.4)	
6a			1.81, m	
6b			1.61, m	
7a		7a, 2.16, m	1.20, m	7a, 1.93, m
7b		7b, 1.40, m	2.19, m	7b, 1.40, m
8	7.41, d (8.4)	8a, 1.66, m	2.43, s	8a, 1.61, m
		8b, 1.71, m		8b, 1.63, m
9	8.05, d (8.4)	1.46, m	1.83, d (12.6)	1.43, m
10		2.00, m		2.47, m
11a	3.75, h (6.6)	2.08, m	2.80, br.d, (12.6)	2.14, m
11b			2.62, br.t, (12.6)	
12	1.38, d (6.6)	0.99, d (6.0)	7.95, s	1.00, d (6.6)
13	1.38, d (6.6)	0.75, d (6.0)		0.94, d (6.6)
14	5.01, s	1.05, d (6.0)	7.74, d (6.0)	1.05, d (6.6)
15	2.54, s		6.37, d (6.0)	
18			0.82, s	
19			0.84, s	
20			0.90, s	

**Table 2:** The ^13^C-NMR (150 MHz) spectroscopic data for compounds **1**–**3** and **6**.

No.	1(MeOD)	2(CDCl_3_)	3(CDCl_3_)	6(CDCl_3_)
	δ_C_	δ_C_	δ_C_	δ_C_
1	124.1, CH	26.3, CH_2_	39.2, CH_2_	25.6, CH_2_
2	136.3, C	35.4, CH_2_	18.9, CH_2_	35.1, CH_2_
3	128.7, CH	199.9, C	42.1, CH_2_	200.9, C
4	122.4, CH	122.5,CH	33.6, C	122.9,CH
5	135.8, C	170.1, C	56.4, CH	168.2, C
6	145.4, C	76.7, C	19.3, CH_2_	76.0, C
7	131.6, C	37.9, CH_2_	29.6, CH_2_	33.7, CH_2_
8	125.6, CH	31.1, CH_2_	39.9, CH	29.7, CH_2_
9	125.7, CH	39.1, CH	52.7, CH	38.2, CH
10	133.4, C	42.0, CH	39.1, C	40.9, CH
11	29.7, CH	30.7, CH	22.7, CH_2_	32.9, CH
12	24.2, CH_3_	15.5, CH_3_	155.0, CH	16.9, CH_3_
13	24.2, CH_3_	16.4, CH_3_	129.9, C	18.0, CH_3_
14	63.8, CH_2_	20.1, CH_3_	155.4, CH	20.3, CH_3_
15	22.2, CH_3_		117.0, CH	
16			179.7, C	
17			179.7, C	
18			33.6, CH_3_	
19			21.6, CH_3_	
20			14.4, CH_3_	

Compound **3** was isolated as a white powder, and the molecular formula was established as C_20_H_28_O_4_ by HR-ESI-MS, which displayed a quasi-molecular ion peak at *m*/*z* 355.1885 [M+Na]^+^. The IR spectrum indicated the presence of methyl (2920 cm^−1^), lactone (1703 cm^−1^), carboxyl (1694 cm^−1^) and double bond (1644 and 1597 cm^−1^) functions. The NMR and HMQC spectra (Table 1 and Table 2) showed the presence of 20 carbons corresponding to two carbonyls [δ_C_ 179.68 (C-16, 17 overlapped)], four olefinic carbons [δ_C_ 155.03 (C-12), 129.89 (C-13), 155.42 (C-14) and 117.02 (C-15)], three methyl groups [δ_C_ 33.60 (C-18), 21.63 (C-19) and 14.35 (C-20)], six methylenes [δ_C_ 39.19 (C-1), 18.88 (C-2), 42.08 (C-3), 19.34 (C-6), 29.64 (C-7), 39.88 (C-8) and 22.70 (C-11)], three methines [δ_C_ 56.36 (C-5), 39.88 (C-8) and 52.68 (C-9)], and two quaternary carbons [δ_C_ 33.57 (C-4) and 39.12 (C-10)]. Analysis of the ^1^H ^1^H-COSY confirmed three isolated proton spin-systems corresponding to H-1–H-3, H-5–H-6–H-7–H-8–H-9–H-11–H-12, and H-14–H-15 units, and the remaining connection was established by detailed analysis of HMBC correlations. In the HMBC correlations (Figure 2), the long-range correlations from CH_3_-20 to C-1, C-5, C-9 and C-10, from CH_3_-18 and CH_3_-19 to C-3, C-4 and C-5 confirmed that C-1, C-5, C-9 and C-20 were connected with C-10, and C-3, C-5, C-18 and C-19 were connected with C-4, respectively. The correlations from H-7, H-8 and H-9 to C-17 determined the connectivity of carboxyl group (C-17) with C-8; The HMBC correlations of H-12 with C-13, C-14 and C-16, and the correlations of H-15 with C-16 implied that a furan-2(3*H*)-one ring was connected with C-12 by the olefinic C-13. Thus the planar structure of **3** was determined.

**Figure 3:**
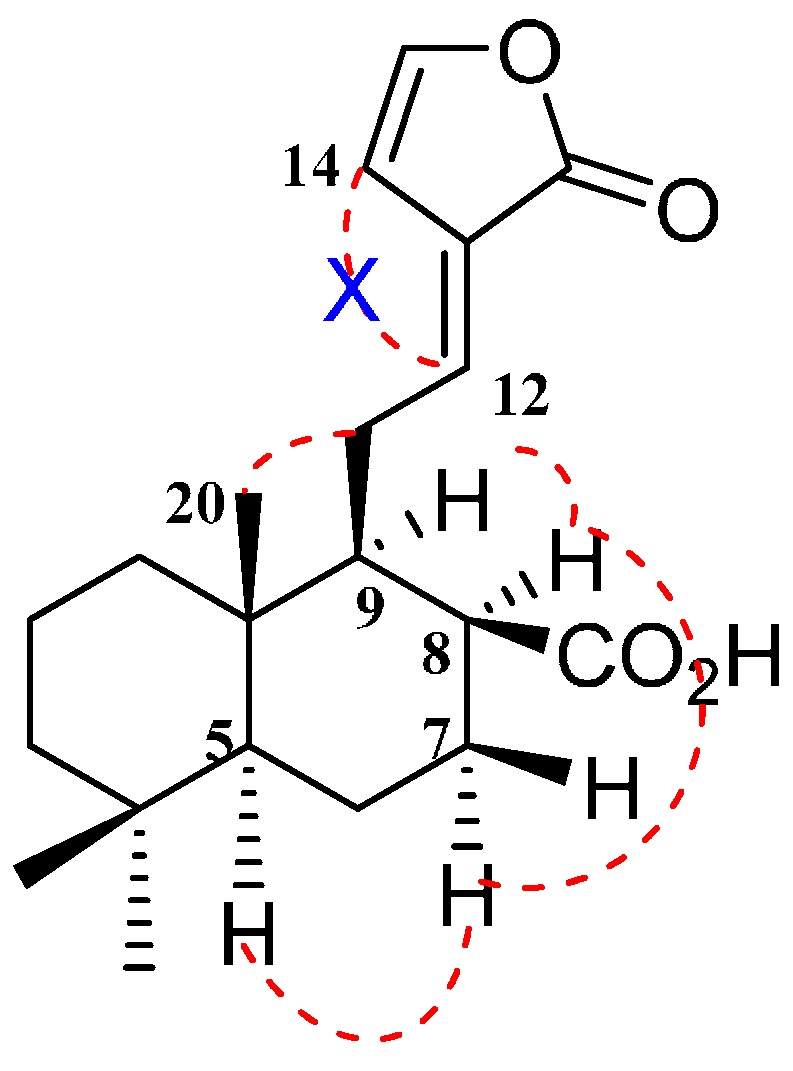
Key NOESY correlations of compound **3**.

The relative configuration of **3** was established by NOESY correlations. The geometry between C-12 and C-13 was *E* configuration, as indicated by a downfield shift observed for H-12 and the absence of a NOESY correlation between H-12 and H-14. The correlations of H-5 with H-7a, H-8 with H-7a and H-9 indicated the α-configuration of these protons, whereas the correlations between Me-20 with H-11 implied that Me-20 and C-11 possessed the β-configuration (Figure 3). Thus, compound **3** was determined to be (*E*)-labda-12,14-dien-15(16)-olide-17-oic acid as shown in Figure 1. This compound was similar to (*E*)-labda-8(17),12,14-trien-15(16)-olide isolated from *Zingiber ottensii* (Zingiberaceae) [16]. The two eremophilane-type sesquiterpenes **4** and **5** were determined to be new natural products, previously identified among transformation products of nootkatone by several different fungi [7,8].

Compounds **1**–**12** were evaluated for the inhibitory bioactivities against glucosidase. Only did compounds **1**, **3** and **6** show moderate bioactive effects with inhibitory rate at 10.3%, 10.0% and 11.5%, respectively, compared to the positive control acarbose at 41.9% at 20 µg/mL.

## 3. Experimental Section

### 3.1. General Experimental Procedures

Optical rotations were measured on a Perkin-Elmer 341 polarimeter. UV spectra were obtained on a Shimadzu UV-2550 visibl spectrophotometer. IR spectra were recorded on a Shimadzu FTIR-8400S infrared spectrometer with KBr disks. HR-ESI-MS were obtained on a LTQ-Obitrap XL LC-MS spectrometer. NMR spectra were recorded on a Bruker Avance DRX-600 instrument at 600 MHz (^1^H-) and 150 MHz (^13^C-), with TMS as the internal standard. CD spectra were recorded on a JASCO J-815 spectropolarimeter, using CH_3_OH as solvent. Purification were performed by Semi-prep-HPLC (Waters 2535 Pump, 2998 Detector). Silica gel H for column chromatography (CC) and silica gel GF254 for preparative TLC were obtained from Qingdao Marine Chemical Factory (Qingdao, China). Precoated plates of silica gel GF254 were used for TLC, and detected under UV light at 254/360 nm.

### 3.2. Plant Material

The fruits of *Alpinia oxyphylla* were collected in Anguo, (Hebei Province, China), in September 2009, and identified by Prof. Baolin Guo, Institute of Medicinal Plant Development, and Chinese Academy of Medical Sciences & Peking Union Medical College. A voucher specimen has been deposited in the Institute of Medicinal Plant Development, Chinese Academy of Medical Sciences & Peking Union Medical College.

### 3.3. Extraction and Isolation

The dried fruits of *Alpinia oxyphylla* (89 kg) were percolated with 95% ethanol (890 L × 2 times) and concentrated under reduced pressure. The residue (10.3 kg) was suspended in H_2_O, and then extracted with *n*-hexane, EtOAc and *n*-BuOH to give 2.2 kg, 2.4 kg and 0.5 kg extracts, respectively. The EtOAc extract (178 g) was subjected to MCI column chromatography eluted with CH_3_OH–H_2_O in a gradient (3:7 to 1:0), to afford 27 fractions (Fr. 1-27). Fr. 14 was eluted with MeOH–H_2_O (1:0–0:1) on a ODS chromatograph, Fr. 14-7 was further separated by preparative HPLC using MeOH–H_2_O (36:64) as the mobile phase to give **7** (6 mg), and Fr. 14-12 to give **8** (17 mg) using MeOH–H_2_O (35:65). Fr. 18 was eluted with MeOH–H_2_O on a ODS chromatograph, Fr 18-2 was further separated by preparative HPLC using MeOH–H_2_O (3:7) as the mobile phase to give **4** (80 mg); Fr. 18-3 to give **11** (28 mg) using MeOH–H_2_O (37:63); Fr. 18-4 was eluted with MeOH on a Sephadex LH-20 chromatograph to give **12** (26 mg); Fr. 18-5 was further separated by preparative HPLC using MeOH–H_2_O (42:58) as the mobile phase to give **5** (31 mg), **9** (21 mg) and **10** (21 mg), and Fr. 18-10 to give **2** (45 mg) and **6** (40 mg) using MeOH–H_2_O (43:57). Fr. 23-24 was eluted with a gradient of petroleum ether–EtOAc (1:0–0:1) on a silica gel H CC, Fr. (23-24)-2 was further separated by preparative HPLC using MeOH–H_2_O (6:4) as the mobile phase to give **3** (10 mg), and Fr. (23-24)-12 to give **1** (4 mg) using MeOH–H_2_O (63:37).

*2-Methyl-6-isopropyl-7-hydroxymethyl naphthalene* (**1**), pale yellow oil, UV (MeOH) λmax: 224 nm, IR (KBr) νmax: 3386, 2961, 2927, 1648, 1458, 1383, 1362 and 837 cm^−1^. HR-EI-MS *m*/*z*: 214.1347 [M]^+^ (calcd for C_15_H_18_O, 214.1357). ^1^H- (MeOD, 600 MHz) and ^13^C- (MeOD, 150 MHz) NMR data see Table 1.

*Oxyphyllenone H* (**2**), colorless oil, $\left[\alpha_{D}^{20}\right]$ −16.67° (c 0.015, MeOH), UV (MeOH) λmax: 241 nm, IR (KBr) νmax: 3451, 2959, 2932, 1661, 1651 and 1021 cm^−1^. HR-ESI-MS(+)*m*/*z*: 223.1682 [M+H]^+^(calcd for C_14_H_2__3_O_2_, 223.1697). ^1^H- (CDCl_3_, 600 MHz) and ^13^C- (CDCl_3_, 150 MHz) NMR data see Table 1.

(*E*)-Labda-12,14-dien-15(16)-olide-17-oic acid (**3**), white powder, $\left[\alpha_{D}^{20}\right]$ +72° (c 0.0075, MeOH), UV (MeOH) λmax: 253 nm, IR (KBr) νmax: 2920, 1703, 1694, 1644 and 1597 cm^−1^. HR-ESI-MS (+) *m*/*z*: 355.1885 [M+Na]^+^ (calcd for C_20_H_28_O_4_Na, 355.1885. ^1^H- (CDCl_3_, 600 MHz) and ^13^C- (CDCl_3_, 150 MHz) NMR data see Table 1.

### 3.4. Inhibitory Activity on Glucosidase

The bioactive assay experiment of inhibitory activity on glucosidase was determined according to the method of reference [17].

## 4. Conclusions

This study reported four new compounds and two new natural products together with six known compounds isolated from the ethyl acetate part of 95% ethanol extract of *Alpinia oxyphylla*. Inhibitory effects of compounds on glucosidase were evaluated, and compounds **1**, **3** and **6** showed moderate bioactivity compared to the positive control acarbose at 20 µg/mL.
